# Memory effects of sedative drugs in children and adolescents—protocol for a systematic review

**DOI:** 10.1186/s13643-016-0192-x

**Published:** 2016-02-18

**Authors:** Karolline A. Viana, Anelise Daher, Lucianne C. Maia, Paulo S. Costa, Carolina C. Martins, Saul M. Paiva, Luciane R. Costa

**Affiliations:** Dentistry Graduate Program, Universidade Federal de Goiás (UFG), Primeira Avenida, s/n, esquina com Praça Universitária, Setor Leste Universitário, CEP: 74605-220 Goiânia, GO Brazil; Department of Pediatric Dentistry and Orthodontic, Universidade Federal do Rio de Janeiro, Cidade Universitária, CCS, Rio de Janeiro, 21941-971 RJ Brazil; Department of Pediatrics, Faculdade de Medicina, UFG, Primeira Avenida, s/n, esquina com Praça Universitária, Setor Leste Universitário, CEP: 74605-220 Goiânia, GO Brazil; Department of Pediatric Dentistry and Orthodontics, Faculdade de Odontologia, Universidade Federal de Minas Gerais (UFMG), CEP: 31270-901 Belo Horizonte, MG Brazil; Division of Paediatric Dentistry, Faculdade de Odontologia, UFG, Primeira Avenida, s/n, esquina com Praça Universitária, Setor Leste Universitário, Goiânia, 74605-020 GO Brazil

**Keywords:** Conscious sedation, Amnesia, Memory, Systematic review

## Abstract

**Background:**

Some sedatives used in children and adolescents can affect memory function. Memory impairment of traumatic experience can minimize the chance of future psychological trauma. Knowledge about the potential of different sedatives to produce amnesia can help in the decision-making process of choosing a sedative regimen. The aim of this systematic review is to evaluate the effect of different sedatives on memory of perioperative events in children and adolescents.

**Methods/design:**

Electronic databases and other sources, such as trial registers, gray literature, and conference abstracts will be searched. Randomized controlled trials will be included that assess memory of perioperative events in children and adolescents 2–19 years old receiving sedative drugs as premedication or as agents for procedural sedation in a medical or dental settings. The outcomes will be loss of memory after and before sedative administration (anterograde and retrograde amnesia). Two independent reviewers will perform screening, study selection, and data extraction. Disagreement at all levels will be resolved by consensus or by involving a third reviewer. Assessment of the risk of bias of included studies will be performed according to “Cochrane Collaboration’s Tool for Assessing Risk of Bias in Randomized Trials.” Clinical and methodological heterogeneity across studies will be evaluated to determine if it is possible to combine or not combine study results in a meta-analysis.

**Discussion:**

To the best of our knowledge, there is no systematic review that specifically addresses this question. Findings from the review will be useful in the decision-making process about the best sedative for minimizing recall of the medical/dental event and possible psychological trauma.

**Systematic review registration:**

PROSPERO CRD42015017559

**Electronic supplementary material:**

The online version of this article (doi:10.1186/s13643-016-0192-x) contains supplementary material, which is available to authorized users.

## Background

Children and adolescents can fear health procedures. In one group of 241 children ranging from 5–12 years of age, 18 % exhibited high preoperative anxiety prior to elective medical surgery under general anesthesia [1]. Prevalence of dental fear and anxiety in children and adolescents is around 11 % [2]. In those cases, sedatives can be used to reduce anxiety.

Sedation is a method for controlling anxiety and behavior, promoting patient safety and welfare, reducing physical discomfort, maximizing the potential for amnesia, and minimizing psychological trauma [3]. The same sedatives used as agents for procedural sedation can be used as a premedication (that is, prior to general anesthesia) with similar aims of sedation [1, 4]. Other benefits of premedication are preventing nausea/vomiting after surgery and reducing postoperative negative psychological effects, such as sleeping problems and negative behavioral changes [1, 4, 5].

When the aim is moderate (formerly conscious) sedation, some sort of crying and movement can persist. In fact, it has been shown that procedural sedation failure ranges from 2.3 % [6] to 66.7 % [7] in children and adolescents. Thus, especially in cases where the child presents behavior management problems despite the sedative, amnesia could be beneficial. Sedation-related amnestic effects refer to the inability to remember experiencing procedures and are a desirable effect. The less a child remembers of a perioperative event, the less likely it is that the child will experience psychological trauma [8]. Furthermore, fewer avoidant behaviors with aversive clinical reactions could be expected from a child that had no memory of traumatic clinical procedures [9]. Psychological trauma in childhood has to be avoided because it can result in neurostructural changes, affect cognitive performance and functioning, and increase the risk of developing psychological disorders [10, 11].

There are some randomized controlled trials analyzing amnesia associated with sedatives as a primary or secondary outcome. During moderate sedation, favorable results have been reported for intranasal midazolam compared to oral hydroxyzine [12], as well as oral ketamine compared to oral dexmedetomidine [13]. Furthermore, midazolam as a premedication has been reported to promote satisfactory anterograde amnesia for information presented after the administration of the sedative and before the induction of general anesthesia [8, 14–16].

To the best of our knowledge, there is no systematic review to specifically address the effect of sedatives on memory. If we have evidence of amnesia related to some sedatives, we are assured that if children and adolescents show distress reactions during procedural sedation or prior to general anesthesia, they will not register it as a negative experience and probably will have a lower likelihood of future psychological trauma.

The aim of this systematic review is to search for scientific evidence of the following question: what is the effect of different sedatives on memory of perioperative events in children and adolescents?

## Methods/design

This protocol was written in accordance to the Preferred Reporting Items for Systematic Reviews and Meta-Analysis Protocols 2015 (PRISMA-P 2015)- Additional file 1 [17]. The steps below will be followed, and differences between the protocol and the review will be reported and accompanied by a description and rationale of the change. The proposed review is registered in the PROSPERO database (CRD42015017559).

### Eligibility criteria

To be included, studies have to follow the criteria outlined below.

#### Types of studies

Randomized controlled trials (RCT), without date and language restriction.

The exclusion criteria will be non-controlled studies, letters to the editor, case reports, in vitro studies, animal studies, observational studies, reviews, conference consensus, and guidelines.

#### Types of participants

Studies that included children and adolescents aged 2–19 years old, without cognitive or neurological impairment, or receiving sedatives as premedication or as agents for procedural sedation in a medical or dental setting will be used in this analysis.

#### Types of intervention and comparison

Studies that evaluated any sedative regimen administered by a health professional in an outpatient setting, dental office, or operation room will be included. The investigated intervention must be compared with placebo, variations of the same sedative regimen (dosage, route, and timing of administration), or with an alternative sedation regimen.

#### Outcomes

The primary outcome will be loss of memory after sedative administration (anterograde amnesia). All types of memory (sensory, short-term, and long-term) will be considered. The secondary outcome will be loss of memory before sedative administration (retrograde amnesia). There are several tasks to evaluate memory, but, in general, the results are the mean number of pictorial stimuli/events correctly recalled or recognized, the number of patients who had any recall, etc.

### Data sources

Searches will be performed using multiple electronic databases, including PubMed, Scopus, The Cochrane Library, LILACS/BBO, CINAHL, Web of Science, Embase, and PsycINFO. We will hand-search the reference lists of all primary studies included. The abstracts of the annual “Pediatric Sedation Outside of the Operating Room Conference” will be searched, and the authors of relevant abstracts will be contacted for further information. To locate unpublished and ongoing trials, we will perform searches in Current Controlled Trials, US National Institutes of Health, the Brazilian Clinical Trials Registry (*Registro Brasileiro de Ensaios Clínicos* (*ReBEC*)), and UK National Institute for Health and Care Excellence. Gray literature will also be searched using OpenGrey, ProQuest dissertations, and the databases “Theses full text” and “*Periódicos Capes*” from the Brazilian agency Coordination for the Improvement of Higher Education Personnel (*Coordenação de Aperfeiçoamento de Pessoal de Nível Superior* (*CAPES*)).

### Search strategy

A search strategy to identify relevant studies was developed under the guidance of a librarian. The descriptors were selected from a controlled vocabulary (Medical Subject Headings (MeSH) and Health Sciences Descriptors (DeCS)), synonymous, related terms, and free terms related to child, adolescent, different sedatives, and memory types. These terms were combined with Boolean operators to allow for a systematic search on the field *Title/Abstract*. Syntax rules of each database were observed. The final search strategy was peer reviewed to check for errors. A PubMed search strategy is included in the Additional file 2. The other search protocols will be revealed in a transparent and reproducible manner.

### Study selection

The reference software program EndNote^®^ (EndNote X7, Thomson Reuters, New York, USA) will be used to organize studies and remove duplicate references. As a training and calibration exercise, two reviewers (KAV and AD) will apply the eligibility criteria to 10 % of the retrieved studies to pilot screening questions and determine inter-examiner agreement. The two reviewers will discuss the disagreements with the supervision of a gold standard (LRC). After achievement of adequate agreement (Kappa 0.72 to 0.77), the two independent researchers will screen titles and abstracts of the studies. In cases of disagreement, the full text will be read. Full text of the articles selected in this preliminary stage will be assessed based on eligibility criteria by two independent reviewers. Any discrepancies will be discussed and resolved by consensus; if necessary, a third reviewer (gold standard) will be consulted.

If the information in the title, abstract, or full text are insufficient or unavailable for making a decision about its inclusion, we will attempt to get the needed information by email sent to the authors (maximum of three times at a 1-week interval). If we cannot obtain an answer, the study will be excluded and listed in the category of “potentially relevant studies.”

### Data collection process

Two authors will independently use a standardized data collection. This form will be pretested using three trials and, if necessary, refined before application in all included studies. Authors will be trained before the data collection process. The following data will be recorded for each included study: article identification (author, year), population (sex and age), number of participants in the group, inclusion and exclusion criteria, sedative regimen (dosage, route, and timing of administration), comparative group, setting, treatment performed, type of memory, measurement methods for memory, statistical techniques used, and results of study analysis. Disagreement will be solved by consensus or, if necessary, by involving a third person (LCM). We will contact study authors using, at most, three attempts to resolve any uncertainties. The reasons for excluding trials will be recorded. If multiple reports of a single study are found, reviewers will evaluate which study will be considered, according to the sample size and outcome.

### Assessment of risk of bias in included studies

Two independent reviewers will undertake the risk of bias assessment of the included studies according to the Cochrane Collaboration’s Tool for Assessing Risk of Bias in Randomized Trials [18]. It addresses seven specific domains concerning five types of bias (selection, performance, attrition, detection, and reporting bias): random sequence generation, allocation concealment, blinding of participants and personnel, blinding of outcome assessment, incomplete outcome data, selective reporting, and other biases. Each potential source of bias will be graded as high, low, or unclear based on criteria for judging the risk of bias [18]. In cases of disagreement, resolution will be attempted by consensus or by involving a third reviewer.

### Data synthesis

Significant heterogeneity, such as sedative regimen, memory type, and measurement methods of memory are expected, which can preclude meta-analyses. However, we will consider clinical and methodological heterogeneity to combine or not combine study results in a meta-analysis. Clinical heterogeneity will be assessed using information about sample, setting, intervention received, and type of memory. Methodological heterogeneity will be assessed using information about the study design and measurement method of memory. Statistical heterogeneity will be evaluated, if possible, by Higgins and Thompson’s *I*^2^ and chi-squared statistics.

It is anticipated that the loss of memory may be reported as categorical or as continuous data. If the meta-analysis is able to be conducted for dichotomous data, calculating the risk ratios along with 95 % confidence intervals is planned, whereas for continuous data, the outcomes will be reported as mean differences if all studies use the same scale or as standard mean differences with corresponding 95 % confidence intervals. If enough data are available, subgroup analyses will be considered according to the different cognitive abilities between age groups [19]: 2–7 years exclusively, 7–12 years exclusively, and 12–19 years exclusively.

The narrative synthesis will be summarized in tables and in the text. It will be guided by the following four main elements, as described by Popay et al. [20]: developing a theory of how the intervention works (why and for whom), developing a preliminary synthesis of findings of included studies, exploring relationships in the data, and assessing the robustness of the synthesis. Additionally, clinical and research implications will be provided.

### Risk of bias across studies

If there are at least ten studies included in the meta-analysis, we will perform funnel plots to assess for potential for publication bias and small study effects. However, if it is not possible, this aspect will be analyzed qualitatively by comparing the results of the studies (presence of significant and non-significant outcomes) and, when possible, comparing the protocol of the RCT with the published study.

We plan to use the Grading of Recommendations Assessment, Development and Evaluation (GRADE) approach to judge the quality of evidence for all outcomes [21]. This approach considers the following aspects to rate the quality of a body of evidence: study design, risk of bias, imprecision, inconsistency, indirectness, and magnitude or effect. Quality will be considered as high, moderate, low, or very low.

## Discussion

This systematic review will synthesize scientific evidence for the effect of different sedatives on the memory of perioperative events in children and adolescents. To our knowledge, this synthesis has not been done yet. It will only take into account randomized controlled trials, which have a higher scientific evidence level when compared with other study designs. The risk of bias for each included study will be evaluated using a methodological quality assessment. Although clinical and methodological heterogeneity across studies may preclude meta-analyses, qualitative narrative synthesis through a systematic review can outline the effect of different sedatives on memory. These results will be valuable for research because if we can find gaps in the literature, this knowledge can inform future research directions in this area. Furthermore, the findings from this systematic review will be valuable in the decision-making process about the best sedative for minimizing recall of the medical/dental event and possible psychological trauma, which can improve patients’ quality of life and the professional practice as a whole.

## Additional files

Additional file 1:
**PRISMA-P (Preferred Reporting Items for Systematic review and Meta-Analysis Protocols) 2015 checklist.** Recommended items to address in a systematic review protocol. (DOC 83.5 kb) Additional file 2:
**PubMed search strategy.** A search strategy in one database is included. (DOCX 14.7 kb)

## Abbreviations

DeCS: Health Sciences Descriptors
GRADE: Grading of Recommendations Assessment, Development and Evaluation
MeSH: Medical Subject Headings
PRISMA-P 2015: Preferred Reporting Items for Systematic Reviews and Meta-Analyses for Protocols 2015
RCT: randomized controlled trials
