# Supplementary material for: Memory effects of sedative drugs in children and adolescents—protocol for a systematic review
Source: Syst Rev. 2016 Feb 18;5:34. doi: 10.1186/s13643-016-0192-x (PMC4759942; doi:10.1186/s13643-016-0192-x)
Supplement: Additional file 2: — PubMed search strategy. A search strategy in one database is included. (DOCX 14.7 kb) [file 13643_2016_192_MOESM2_ESM.docx]

**Additional file 1 – PubMed Search strategy**

(((((Child [mh] OR Child* [tiab] OR Pediatric[tiab] OR Paediatric[tiab] OR Child, preschool [mh] OR Preschool Child*[tiab] OR Adolescent [mh] OR Adolescen*[tiab] OR Youth*[tiab]))) AND ((Hypnotics and sedatives [mh] OR Hypnotic* [tiab] OR Sedative* [tiab] OR Tranquilizer [tiab] OR Histamine H1 Antagonists [Mesh:NoExp] OR Antagonists Histamine H1 [tiab] OR Antihistamines Classical [tiab] OR Antihistamines Sedating [tiab] OR Tranquilizing agents [Mesh:NoExp] OR Tranquilizing [tiab] OR Narcotics [mh] OR Narcotic*[tiab] OR Anesthetics [Mesh:NoExp] OR Anesthetic*[tiab] OR Anaesthetic*[tiab] OR Analgesics, opioid [mh] OR Opioid*[tiab] OR Anti-anxiety agents [mh] OR Tranquillizing Agents Minor[tiab] OR Anti-Anxiety[tiab] OR Antianxiety[tiab] OR Anxiolytic*[tiab] OR Benzodiazepines [mh] OR Benzodiazepine*[tiab] OR Midazolam [mh] OR Midazolam [tiab] OR Diazepam [mh] OR Diazepam [tiab] OR Nitrous oxide [mh] OR Nitrous oxide [tiab] OR N2O[tiab] OR Relative analgesia[tiab] OR Barbiturates [mh] OR Barbiturates [tiab] OR Anesthetics, Intravenous [mh] OR Intravenous Anesthetics [tiab] OR Anesthetics, Dissociative [mh] OR Anesthetics Dissociative [tiab] OR Dexmedetomidine [mh] OR Dexmedetomidine [tiab] OR Meperidine [mh] OR Meperidine [tiab] OR Pethidine[tiab] OR Fentanyl [mh] OR Fentanyl [tiab] OR Adjuvants, Anesthesia [mh] OR Adjuvants Anesthe* [tiab] OR Morphine [tiab] OR Morphine Derivatives [mh] OR Ketamine [mh] OR Ketamine [tiab] OR Ketorolac Tromethamine [mh] OR Ketorolac Tromethamine [tiab] OR Ketorolac [mh] OR Ketorolac [tiab] OR Indomethacin [mh] OR Indomethacin [tiab] OR Tolmetin [mh] OR Tolmetin [tiab] OR Chloral hydrate [mh] OR Chloral hydrate [tiab] OR Hydroxyzine [mh] OR Hydroxyzine [tiab] OR Promethazine [mh] OR Promethazine [tiab] OR Phenergan[tiab] OR Propofol [mh] OR Propofol [tiab] OR Anesthetics, inhalation [mh] OR Anesthetics inhalation [tiab] OR Gases Anesthetic[tiab] OR Sevoflurane[tiab]))) AND ((Amnesia, anterograde[mh] OR Amnesias Anterograde[tiab] OR Memory Loss Anterograde[tiab] OR Amnesia [Mesh:NoExp] OR Amnesia [tiab] OR Amnesia Global [tiab] OR States Amnestic[tiab] OR Memory Loss[tiab] OR Amnesia, retrograde [mh] OR Amnesias Retrograde[tiab] OR Memory Loss Retrograde [tiab] OR Memory, Episodic [mh] OR Memory Episodic [tiab] OR Memories Episodic[tiab] OR Memories Autobiographical[tiab] OR Memory Autobiographical [tiab] OR Prospective Memory[tiab] OR Memory[Mesh:NoExp] OR Memory [tiab] OR Memory, short-term [mh] OR Short-Term Memory[tiab] OR Working Memory[tiab] OR Sensory Memory[tiab] OR Memory, long-term [mh] OR Memory long-term [tiab] OR Explicit Memory [tiab] OR Declarative Memory[tiab] OR Semantic Memory[tiab] OR Nondeclarative Memory[tiab] OR Procedural Memory[tiab] OR Mental recall [mh] OR Recall Mental[tiab] OR Memory Recall [tiab] OR Memory Retrieval[tiab] OR Recognition Memory[tiab] OR Repetition priming [mh] OR Priming Repetition [tiab] OR Implicit memory[tiab] OR recall [tiab]))) NOT ((case reports[Publication Type] OR letters [Publication type] OR editorial[publication Type] OR review[Publication Type]))
